# The Most Common Reasons and Incentives of Tendency to Addiction in Prisons and Rehabilitation Centres of Zahedan (Iran)

**DOI:** 10.5539/gjhs.v7n4p329

**Published:** 2015-01-25

**Authors:** Ahmadali Raeisei, Hamed Sarani, Azizollah Arbabisarjou, Azizollah Mojahed

**Affiliations:** 1 Department of Cardiology, Zahedan University of Medical Sciences, Zahedan, IR Iran; 2 Pregnancy Health Research Center, Zahedan University of Medical Sciences, Zahedan, IR Iran; 3 Department of Psychology, Zahedan University of Medical Sciences, Zahedan, IR Iran

**Keywords:** addiction, addicted, prison, rehabilitation

## Abstract

In most European countries the ratio of addicts and normal people is 1 to 5,000, and in some third world countries such as Morocco, Egypt, and South Africa this figure is 1 to 1,000. In Iran, the situation is worse, and there is one drug addict per 100 people. The research method in this study is analytical descriptive. The study population consisted of all rehabilitated addicted men and women who were spending their time in prison. Total sample size were 134 people (99 men and 35 women).

A special designed questionnaire used to collect data, which included socio-demographic characteristics. The validity of the questionnaire has content validity and for reliability, Cronbach α was used which was 0.78. The formal years of education was 4.3 year. The average age of the first drug use was 12 years for men and 22 years for women. Sixty-nine point seven percent of men used drugs with their friends and 31.4% of women used drug with their husbands. The men more than women, single men more than married women, and illiterates or poor literates were more at risk. Most men blamed bad friends and women blamed physical and psychological problems as the cause of addiction. The singles got addicted due to bad friends and married individuals were addicted due to emotional distress. The majority of age group (27-13 years), got addicted due to bad friends and older groups addicted due to emotional distress. In other words, the older the person gets, the influence of bad friends decreases, and the effect of psychological distress due to conflict and adversity increases.

## 1. Introduction

Today, the problem of addiction extends beyond the boundaries of health and treatment and turned into a social crisis and an ominous phenomenon. Drug abuse is one of the most controversial issues in the field of psychology and sociology that attracted experts. So that it can be said with certainty that increasing use of drugs has been turned into one of the large and complex problems in human societies. This complexity arises because addition is a biological, mental, sociological, economic, and cultural problem and it could not be paid attention only from one angle (Aghabakhshi et al., 2009). As experience has shown that no success has been achieved with this view. In other words, drug addiction as one of the greatest challenges in human populations, has a lot of hidden and evident dimensions and angles (Habibi et al., 2012).

Addiction is a behavioural habit which its abandonment is often difficult. Addiction could be to alcohol, drugs or gambling. Many of us seek shelter to addiction to deal with the problems of life. Use of alcohol, drugs or gambling relieves pain or fear for a while. But in the end, it itself becomes a problem, which breaks apart most relationships and families (Momtazi, 2002; Rastegar, 2002). Around the world, the number of drug addicts reaches to 190 million persons and official records in Iran state the number of addicts 1.2 to 2 million whose mean age is18 years, whereas the 11 million people are dealing with their addiction or addiction of their relatives (Shafiei et al., 2004; Ziyaaldin et al, 2006). Addiction in Iran has shown the annual growth of 8 percent over the past 30 years (National Study Centre on Addiction, 2007). Deputy of Ministry of Health in an interview with Fars social reporter stated that the total number of drug abusers in Iran is three million and 700 thousand. Of them, one million and 200 thousand people are addicted and use every day and the rest use it for recreation or take psychedelic drugs. About 60 percent of the prison space is occupied by prisoners convicted with addiction (Ghaemi, 1997). In year 2000, about 243 thousand people have been arrested in connection with drugs which 93% of them belonged to minor drug traffickers or addicts. The country’s annual consumption of drug is 780 tons and drug users smoke more than 225 billion toman worth of drugs. Young people are the main victims of drug abuse. Many studies have shown that culture, social and familial issues are closely related to addiction, in such a way that determines the social standard of how anyone reacts to any phenomenon (Maddux & Desmond, 1979). For example, in the United States the use of alcohol in families whose parents are consuming alcohol, starts early in their teenagers (Spiro, 1955). And in groups that are under the control of alcohol consumption, alcoholism is less common and in groups that alcohol is consuming easily, alcoholism is more prevalent. Recent researches have shown that juvenile delinquent, addicts, or runaways are children of divorce or came from crowded and poor families who were cheated by drug traffickers or abused at brothels. They are between 12 to 30 years old and this phenomenon is growing every day. Other issues like shortage of cultural, sport, and recreational facilities have increased the risk of young people tendency towards drugs, in as much as in deprived border lands the situation is more acute and using drugs between young people and together at homes has turned into a normality. Approximately 90% of murders, robberies, rapes of women and girls and human trafficking and the prevalence of AIDS are directly or indirectly related to drugs. According to a study by Iran Drug Control Headquarter in 1998, the students in Yazd province with respect to of drug exposure were in fourth place among the country provinces (Shafiei et al., 2004).

In addition to the increasing prevalence of substance-use, unfortunately age of onset is decreasing too, so that according to the study of Berjas et al, the lowest age of onset of addiction was 9 years old and among various personal, family and social factors, the imitation of the addicted adults, parents addiction and interaction with drug abusers had the most effect on tendency to drug use, respectively (Berjas et al, 2011). Numerous studies have cited various reasons for addiction. In the study of Rahimi et al. most of women stated social remark, humiliation and withdrawal by family and society as well as severe poverty as their most important problems (Rahimi et al. 2011), but with regard to multifactorial nature of behaviour, one factor does not affect the addiction alone, as in the study of Sharg et al, the findings showed that a set of individual, familial, social and economic causes with different proportions causes relapse (Sharg et al., 2011), indicating the need for complete and accurate understanding of the factors and designing studies with a focus on relapse factors and approaches to prevent that. According to the researches of Nastizayi et al. (2010), it was determined that factors such as corrupted environment, addicted friends, ineffective psychotherapy sessions and association of ideas are involved in addiction relapse. Also, the effect of drug relapse (corrupted environment, addicted friends, ineffective psychotherapy sessions, factors of the association of ideas) on sex and age groups are self-introduced addicts were similar. In the study of Seragi et al., (2010), the major causes of person’s addiction is enjoyment and curiosity (75.1%) and the most important factor in a person’s cold turkey was tiresome and family pressure (45.4%). Therefore, with regard to high rate of addiction in the country and its social and health problems, taking addiction as one of the top health priorities of the country which jeopardize the health of the society, seems very reasonable and each society depending on the special features of people, suffer from it in a way. The socio-cultural specifications, attitudes and views of the society especially those in relation with addiction could expose any groups of the society at risk of drug abuse (Seragi et al., 2010).

Most of the existing resources of the country are merely reports about researches and theories which were compiled by foreign researchers and so far no comprehensive research has done about effective factors in addiction relapse followed by returning to care centres (Prison or rehabilitation centres) based on conditions, cultural and economic customs of Iran society in general and Zahedan population in particular.

### 1.1 Research Questions

- Are socioeconomic and political conditions of community involved in the issue of addiction?
- Are age, gender, performing religious chores, and marital status involved in the issue of addiction?

## 2. Methodology

In this descriptive research is descriptive- analytical, the study population consisted of addicted men of rehabilitation centre and addicted women in prison, in which the sample size comprised 134 participants, including 99 men and 35 women. There are other people in the prison and rehabilitation centre with convictions other than addiction, but only addiction was taken into account and statistics population consists of only the addicts. A questionnaire included some variables such as age, sex, education, and job was used to collect data. The validity of the questionnaire approved through views of experts in clinical psychology, health education and psychiatrists. For determining the reliability, Cronbach α was used which it obtained 0.87. After the data were collected, they were coded and entered into the computer and analysed using statistical software SPSS 18. The study proposal has reviews and approved by Research Council of Zahedan University of Medical Sciences, Iran.

## 3. Research Findings

Table 1 shows 73.9% are men and 26.1% are women among addicts, and the results showed that the illiteracy rate for women is higher than men.

**Table 1:** Relative frequency distribution of addicts in terms of gender, age and educational level

Statistical Indexes	13-27 y	28-42 y	43-57 y	58-72 y	>72 y
Men	(74.0)	19%	5%	1%	-
Women	48.6%	34.3%	17.1%	-	-
Total	69.7%	23.1%	8.2%	0.7%	-
Gender and Education	Illiterate	Elementary School	Guidance School	High School	Higher than High School
Men	29.2%	35.8%	22.9%	11.1%	1%
Women	50%	32.8%	2.9%	14.3%	-
Total	34.3%	35.1%	17.9%	11.9%	0.7%

Most of the family members of addicted persons in women were between 1 and 5 people but in men there were 6 to 10 people (Table 2).

**Table 2:** Relative frequency distribution of the number of family members of addicts before drug addiction

Gender and No. of family members	1-5 persons	6-10 persons	>11 persons
Men	39.4%	46.5%	14.1%
Women	82.9%	17.1%	-
Total	50.7%	38.8%	10.4%

With regard to the results in Table 3, lack of interest in studying about addiction (22.3%) is the most frequent reason for dropout of addicts in men, and the next is the need for substance (16.4%). For the women, need to substance (30.7%) is the first reason and then lack of parents’ interest to with drawl (23%).

**Table 3:** Relative frequency distribution of reasons for dropouts of addicts with respect to sex

Reasons for dropouts and sex	Men	Women	Total
No interest in studying	22.3%	-	18.5%
Need	16.4%	30.7%	18.5%
Fail	14.9%	-	12.3%
Expulsion	11.9%	-	9.8%
Addiction	8.9%	-	7.4%
Conviction	1.4%	-	1.2%
Sentence	1.4%	-	1.2%
Illness	1.4%	7.4%	2.4%
Parent’s lack of interest	-	23%	3.7%
Others	20.3%	38.4%	23.3%

Worker was the most frequent job among addicts and the second most was driving (Table 4).

**Table 4:** Relative frequency distribution of occupation and gender in studied drug addicts

Gender and addicted member	Father	Mother	Sister	Brother	Spouse	All family	Other relatives
Men	12.5%	12.5%	6.2%	18.7%	6.2%	18.7%	25%
Women	5,2%	5.2%	5.2%	15.7%	42.1%	15.7%	10.9%
Total	8.5%	8.5%	5.7%	17.1%	25.7%	17.1%	17.4%

In men addicted families, the brother is often addicted, and in women addicted families, the husband is often addicted (Table 5).

**Table 5:** Relative frequency distribution of addicted member in studied families of addicts

Gender and Job	Clerk	Worker	Technician	farmer	Driver	False Occupation	Others	Unemployed
Men	16.1%	17.2%	15.2%	8.1%	20%	10.1%	8.1%	15.2%
Women	14.3%	17.1%	0	2.9%	0	0	11.4%	54.3%
Total	8.2%	17.2%	11.2%	6.7%	14.9%	7.5%	9.9%	31.3%

The results of Table 6 indicate that the most frequent cause of addiction in women was mental disorders and in men was “bad friends”.

**Table 6:** Relative frequency distribution of cause of addiction and gender in studied addicts

Gender and cause of addiction	Men	Women	Total
Bad friends	39.4%	14.3%	32.8%
Mental disorders	21.2%	42.8%	27.7%
Addicted family	3%	5.7%	3.7%
Physical problems	4%	28.6%	9%
Unemployment	6.1%	5.7%	6%
Others*	25.3%	2.9%	20.8%

* The subjects did not like or want to write the causes and incentives of addiction.

## 4. Discussion and Conclusion

Seventy-four percent of men were under 27 years old and 48.6% of women were at the same age group. In a similar research that was done in rehabilitation center of Zahedan, the majority of addicted men were between 20 and 40 years old and addicted women were between 10 and 30 years old (Soltani & Saiidpour, 1988). Thirty-four point three percent of all addicts were illiterate. 50% of addicts’ women were illiterate. The literate ones were more completed primary education (35.9%). In general, the average education level was 3to 4 class, which suggests a low level of education in these subjects.

In a similar study in 1988 in Zahedan Rehabilitation Research, this figure was 28% and another research findings demonstrated it 27% (Soltani & Saiidpour, 1988; Soltani, 1990). These figures reflect the fact that addiction prevails mostly in illiterate and poorly literate groups; So we have to focus more on these groups and enhance our educational system for prevention in such a way that people at any age group or everywhere in this country could benefit from further education, considering this fact that addiction in people with higher education is much less than illiterates and other groups. Eighty-three point three percent of addicts have left school. The average age of dropout was 8.7 years. Sixty point four percent have left the school between the ages of 7-11 years. This means that in a year studying at the primary school. Most frequent reason of the dropouts in men was lack of interest and in women was the need. This suggests that the causes of dropouts in two sexes are different and must be examined separately. This means that in men, the interest in study must be developed and this is possible through a variety of trainings that are carried out at the level of the community, neighbourhood and family. But the situation is different for women because they are facing with more problems than men in the society for furthering their education. Economic weakness of the family is an important factor in the failure to complete the girls’ education, and in this regard, something should be done for this problem. For Socio-cultural and poverty, there was no interest in parents to educate girls and foster the attitude that women should stay at home and do housework are the existing problems. As it was seen the second cause of school dropout in women, was parents’ lack of interest in education. A number of studied addicted women have stated that they left the school after the marriage. Low marriage age and also opposition to education after marriage is a problem that must be addressed.

Sixty point six percent of the men were single before addiction and their number has not changed after drug addiction, but the 62.9% of the women were single before addiction and 74.3% were married after addiction. The results showed that men got addicted when they were single but women got addicted after marriage and this might show the mental pressure in the family of addicted women and also this statistics shows that in men the effects of the environment, including bad friends and other conditions but marriage, are more influential in their addiction. In the research by Soltani and Saiidpour which was carried out in Zahedan about rehabilitation of addicts, in 1988, the result showed that single addicted women were half of single addicted men (Soltani and Saiidpour, 1988). It means that addicted women were more married and addicted men were more single. On the other hand, we must pay attention to after marriage factors in the family of women (including, spouse’ addiction, diseases, …) and consider it in this regard.

Men before addiction lived more with father and mother and women lived with their husbands before the addiction. In the study of Kerman, 79% of self-represent addicts lived in father’s house when they started to use drugs (Mansori, 1998).

Eighteen point five percent of studied addicts were not satisfied from their environment and once again unsatisfied women were more than men (34% versus 12.6%). In Kerman research 92% of those who lived with their parent and72% of those who lived with their partners were not satisfied with their family environment. There, in 79% of cases, families of self-introduced addicts were not aware of the activities of their children and this shows that we should not be deceived by the good relationship of parents with children, i.e. having a good relationship could not be an obstacle to addiction (Mansori, 1998).

Maximum age of onset of addiction was 14-26 years (61.9%). This age applies for both men and women. There are a few people that start drugs after 40 years old. In a similar study in rehabilitation centre of Zahedan in 1988, the dominant starting age was 15-30 years. But there was a difference in age of onset of drug abusers, where men started addiction between 20-15 years and women between 30-20 years (Soltani & Saiidpour, 1988). Taken together, these findings suggest that onset of addiction is around youth times, the age at which a person gets ready for a new life. In other words, the person gets married and starts a family, finds a job, and become a part of building the society. But, unfortunately this would not happen and the person with his addiction ruins many years hard work of family, government, and nation.

In general, most addicts blame bad friends as the cause of addiction. However, men and women had different opinions. The bad friends for men, and emotional distress for women are more involved in addiction. In women physical problems and for men mental disorders were in the second place. The impact of unemployment had the lowest percentage (6%). According to obtained information, we must distinguish between men and women and make necessary measures accordingly. In 1990 study in Zahedan, the causes of addiction were considered as follows: 52% unemployment, 25% the abundance of drugs in the market and drug trafficking, and the next factor to consider was cultural factors (Soltani, 1990).

Forty-three point six percent of drug users said that they have spent their free time with doing nothing. But women have spent their leisure time with art (44.1%). Fifteen point eight percent of drug addicts spent their time with getting together, 11.9% with art, 6%, with recreation, 3.8% exercise, 3.8% study. Spending leisure time is an important factor that could affect the propensity to addiction or abandoning it. We see that 43.6% of addicts spend their leisure time with doing nothing. Women also had no entertainment.
